# Efficacy of a multifaceted podiatry intervention to improve balance and prevent falls in older people: study protocol for a randomised trial

**DOI:** 10.1186/1471-2318-8-30

**Published:** 2008-11-25

**Authors:** Martin J Spink, Hylton B Menz, Stephen R Lord

**Affiliations:** 1 Musculoskeletal Research Centre, Faculty of Health Sciences, La Trobe University, Bundoora 3086, Victoria, Australia; 2 Prince of Wales Medical Research Institute, Randwick 2031, New South Wales, Australia

## Abstract

**Background:**

Falls in older people are a major public health problem, with at least one in three people aged over 65 years falling each year. There is increasing evidence that foot problems and inappropriate footwear increase the risk of falls, however no studies have been undertaken to determine whether modifying these risk factors decreases the risk of falling. This article describes the design of a randomised trial to evaluate the efficacy of a multifaceted podiatry intervention to reduce foot pain, improve balance, and reduce falls in older people.

**Methods:**

Three hundred community-dwelling men and women aged 65 years and over with current foot pain and an increased risk of falling will be randomly allocated to a control or intervention group. The "usual cae" control group will receive routine podiatry (i.e. nail care and callus debridement). The intervention group will receive usual care plus a multifaceted podiatry intervention consisting of: (i) prefabricated insoles customised to accommodate plantar lesions; (ii) footwear advice and assistance with the purchase of new footwear if current footwear is inappropriate; (iii) a home-based exercise program to strengthen foot and ankle muscles; and (iv) a falls prevention education booklet. Primary outcome measures will be the number of fallers, number of multiple fallers and the falls rate recorded by a falls diary over a 12 month period. Secondary outcome measures assessed six months after baseline will include the Medical Outcomes Study Short Form 12 (SF-12), the Manchester Foot Pain and Disability Index, the Falls Efficacy Scale International, and a series of balance and functional tests. Data will be analysed using the intention to treat principle.

**Discussion:**

This study is the first randomised trial to evaluate the efficacy of podiatry in improving balance and preventing falls. The trial has been pragmatically designed to ensure that the findings can be generalised to clinical practice. If found to be effective, the multifaceted podiatry intervention will be a unique addition to common falls prevention strategies already in use.

**Trial registration:**

Australian New Zealand Clinical Trials Registry: ACTRN12608000065392

## Background

Falls in older people are a major public health problem, with one in three people aged 65 and over falling each year [1,2]. One-quarter to one-half of all falls among community-dwellers cause some injury, 10–15% of falls are associated with serious injury, 2–6% with fractures and around 1% with hip fractures [3]. The most commonly self-reported injuries include superficial cuts and abrasions, bruises and sprains. The most common injuries that require hospitalisation comprise femoral neck fractures, other fractures of the leg, fractures of radius, ulna and humerus and fractures of the neck and trunk [3]. Falls are the leading cause of injury-related hospital admissions in older people, accounting for 4% of all hospital admissions in this age-group [4].

It is now well recognised that falls result from an interaction between environmental hazards and a broad array of physiologic risk factors, including impaired vision, reduced muscle strength, diminished peripheral sensation and slow reaction time [5]. However, one potentially significant falls risk factor that has only recently been explored is foot impairment. Foot problems affect one in three community dwelling people over the age of 65 years [6,7] and are associated with reduced walking speed and difficulty performing activities of daily living [8-10]. A recent prospective study of 176 older people indicated that ankle flexibility, toe plantarflexor strength and plantar sensation were significant and independent predictors of balance and functional test performance, explaining up to 59% of the variance in these test scores [11]. A 12-month follow-up of this cohort confirmed that these factors, in addition to foot pain, were significant independent predictors of falls [12].

In addition to foot pain and impairment, inappropriate footwear may also play a role in increasing falls risk. A number of studies have assessed footwear in older people who have fallen, and the evidence indicates going barefoot or wearing stockings increases the risk of a fall, as does an increased heel height and smaller sole contact area [13,14]. A number of other studies have investigated the main features of a shoe thought to affect balance, with heel height [15], heel collar height [16], fixation (method used to attach the shoe to the foot) [17] and the slip resistance properties of the sole [18] all being associated. This evidence suggests there is a relationship between footwear and falls and that wearing appropriate footwear may reduce the risk of falls.

Given the emerging evidence that foot problems and inappropriate footwear increase the risk of falls, it has been suggested that podiatry may have a role to play in falls prevention, with several guidelines recommending that older people have their feet and footwear examined by a podiatrist [19-21]. In light of this, it is surprising to find that podiatry has been largely overlooked in falls intervention trials. Only one intervention study has included foot and footwear assessment as part of a nurse-led multifactorial approach to falls. In this study, the assessments were conducted by trained nurses who referred to a relevant specialist depending on the problem found, which in the case of foot problems or inappropriate footwear was a podiatrist [22]. Furthermore, podiatry currently plays a relatively minor role in multidisciplinary falls clinics, with two recent surveys indicating that only 4 out of 25 Australian falls clinics [23] and 1 out of 105 National Health Service trusts in London involved in falls prevention activities [24] utilised podiatrists.

While falls prevention guidelines have recommended that older people have their feet and footwear examined by a podiatrist, these guidelines do not specify the assessment or intervention activities to be undertaken. This may be due to the limited amount of evidence from clinical trials regarding the efficacy of podiatry treatment in reducing pain, improving mobility and decreasing the risk of falls. Although callus debridement and accommodative padding have been shown to reduce weightbearing pressures under the foot [25-27] and reduce pain [28-30], a small pilot study by Balanowski and Flynn is the only study to assess any form of podiatric treatment in relation to functional ability and balance in older people [28]. Functional and static balance tests were conducted before and seven days after scalpel debridement of painful plantar keratoses in 19 older people. Follow-up tests indicated significant improvements in functional ability, however the small sample size, lack of a control group and short follow-up time indicate that further work in this area is required.

An additional factor that has been largely overlooked in the literature is the role of foot and ankle stretching and strengthening in improving balance and decreasing the risk of falls. Although muscle weakness has been shown to be an important risk factor for falls [31] and exercise is recommended in recent evidence-based guidelines for falls prevention [32-34], only one study has demonstrated that increasing toe plantarflexor strength can improve balance ability [35]. Similarly, while reduced ankle flexibility is associated with falls [12], few studies have directly evaluated the potential benefits of ankle stretching and strengthening in improving balance even though it has been shown to improve with stretching [36] and water exercise [37].

Given the detrimental effects of foot problems and inappropriate footwear and the paucity of studies undertaken to ascertain the efficacy of podiatry treatment in falls prevention, the aims of this project are to determine the effectiveness of a multifaceted podiatry intervention in (i) reducing disabling foot pain, (ii) enhancing balance and mobility, and (iii) preventing falls in older people.

## Methods

### Design

**Figure 1:**
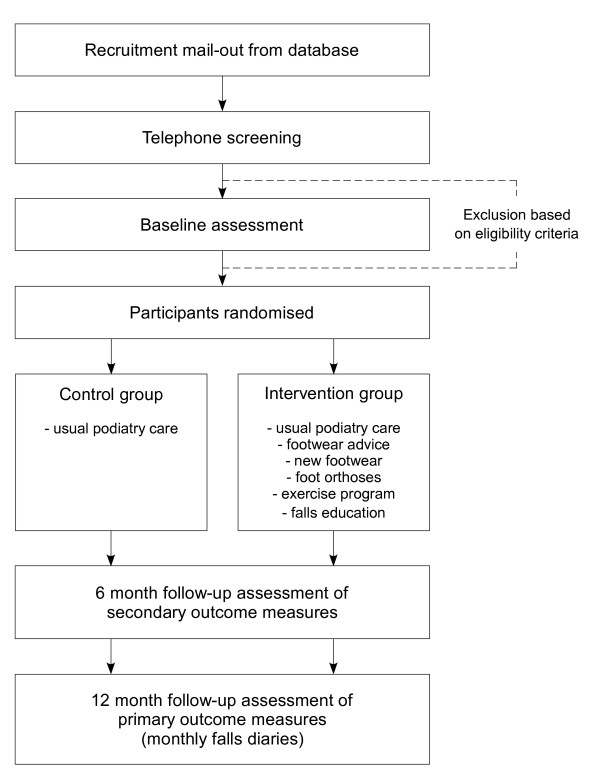
Design of study.

This study is a parallel-group randomised trial with a one-year follow-up (Figure 1). The Human Ethics Committee of La Trobe University Human has approved the trail and all participants will give written informed consent. Participants will be randomly allocated to either a "usual care" control group or the "multifaceted podiatry" intervention group. Permuted block randomisation will be undertaken using an interactive voice response telephone service provided by the National Health and Medical Research Council Clinical Trials Centre at the University of Sydney. Assessors will be blinded to group allocation but due to the nature of the trial, the participants will not be blinded to group allocation.

### Participants

Community dwelling men and women aged 65 years and over will be recruited by a mail-out letter from two databases: (i) people who are currently accessing podiatry services at the La Trobe University Health Sciences Clinic, Bundoora, Victoria, Australia and (ii) people who are currently accessing podiatry services at the Bundoora Extended Care Centre, Victoria, Australia as well as from advertisements placed in seniors newspapers and websites. Respondents will be initially screened by telephone to ensure they are able to walk household distances without the use of a walking aid and are able to read and speak basic English. Individuals who meet the initial screening criteria will then be invited to be assessed for eligibility.

To be included in the study, participants must meet the following inclusion criteria:

(i) an elevated risk of falling, defined as either a history of a fall in the previous 12 months, a score of > 1 on the short Physiological Profile Assessment (PPA) [5] or performance on the alternate stepping test (the time taken to alternately place each foot on a 19 cm high step eight times) of > 10 seconds [38];

(ii) self-reported disabling foot pain (defined as people who have had foot pain lasting for at least a day within the last month and a positive response to at least one item on the Manchester Foot Pain and Disability Index [39]);

(iii) cognitively intact (defined as a score of ≥ 7 on the Short Portable Mental Status Questionnaire [40]).

Participants will be excluded if they have Parkinson's disease (or other neurodegenerative disorders) or lower limb amputation (including partial foot amputation).

### Control group

The control group will be asked to continue whatever podiatry treatment they currently receive for the 12 months of the study. All participants will be offered basic podiatry treatment free of charge in the La Trobe University Health Sciences clinic for the 12 months of the study regardless of whether they are current patients of the clinic. This will typically include toenail maintenance and scalpel debridement of hyperkeratotic lesions (corns and calluses). This intervention is consistent with the usual ongoing "maintenance" care that is provided to older people attending public sector podiatry services (such as community health centres, outpatient podiatry clinics and Department of Veterans' Affairs subsidised private podiatry).

### Intervention group

**Figure 2:**
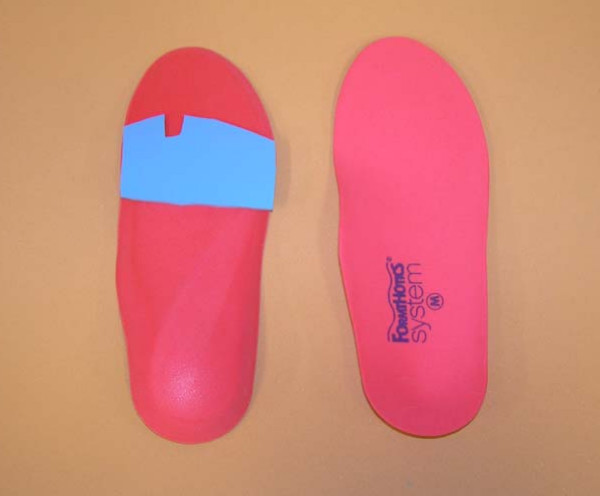
Prefabricated orthoses (Formthotics(tm), Foot Science International Ltd, Christchurch, New Zealand) used in the study.

**Figure 3:**
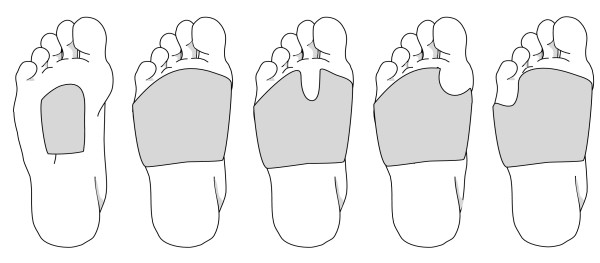
Examples of insole designs to redistribute pressure away from plantar hyperkeratotic lesions to be incorporated into the foot orthoses used in the study.

**Figure 4:**
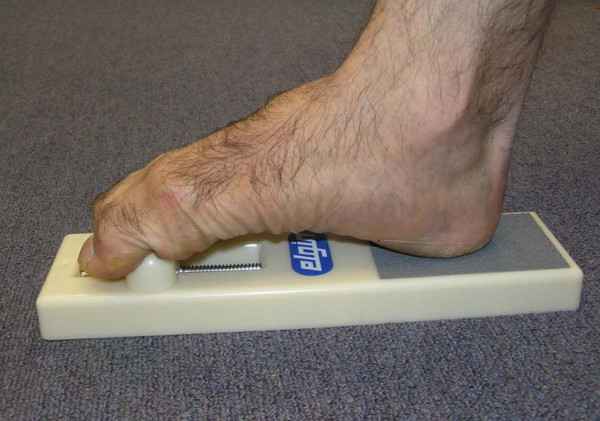
The Archxerciser (Elginex Corporation, Lombard, Illinois, USA) to strengthen toe plantarflexor muscles..

The intervention group will also be asked to continue whatever podiatry treatment they currently receive for the 12 months of the study and all participants will be offered basic podiatry treatment free of charge in the La Trobe University Health Sciences clinic for the 12 months of the study regardless of whether they are current patients of the clinic. In addition, they will receive a multifaceted podiatry intervention consisting of:

(i) *Footwear advice and provision*: participants' outdoor footwear will be assessed using a footwear assessment form for which the component variables have been shown to have good intra-rater reliability (intra-rater kappa 0.62 – 1.0) [41]. Participants will be deemed to have inappropriate footwear if the heel height is greater than 4.5 cm or the shoe has any two of the following; no fixation, no heel counter or the heel counter can be depressed to greater than 45°, the tread pattern of the sole is fully worn or manufactured with a smooth sole, or the shoe heel width is narrower than the participant's heel width by greater than or equal to 20%. Participants with inappropriate footwear will be counselled regarding the specific hazardous footwear feature/s identified, and will be provided with a handout on what constitutes a safe shoe. They will then be given the contact details of an extra-depth and medical grade footwear retailer and will be asked to purchase a more appropriate pair of shoes. Their purchase of footwear will be assisted by the provision of an AUD$100 voucher.

(ii) *Foot orthoses*: Prefabricated insoles (Formthotics™, Foot Science International Ltd, Christchurch, New Zealand) manufactured from a thermoformable cross-linked closed cell polyethylene foam will be shaped to fit the participant's foot (Figure 2). The orthoses will then be appropriately customised using 3 mm thick PPT urethane to redistribute pressure away from plantar lesions (Figure 3).

(iii) *Home-based exercise program*: participants will be provided with instructions and practical demonstrations at the baseline assessment to allow them to undertake a 30 minute home-based exercise program aimed at stretching and strengthening the muscles of the foot and ankle. Strength will be assessed at baseline and appropriate intensity prescribed. A summary of the individual exercises is provided in Table 1. All necessary equipment to undertake the exercise program, including an Archxerciser™ (Elginex Corporation, Lombard, Illonois, USA) (Figure 4) will be provided. Participants will also be given an illustrated explanatory booklet of each exercise, a DVD demonstrating the exercises and an exercise diary to be returned monthly to the researchers. The program will be performed three times per week for six months, and participants will be contacted at one, four, twelve and twenty weeks by telephone to promote adherence to the program.

**Table 1:** Description of specific exercises.

**Name**	**Description**	**Dosage**	**Increments**
Ankle range of motion	Sitting with leg extended. Rotate foot in clockwise direction then anti-clockwise	1 × 10 repetitions for each foot in each direction	None
Ankle dorsiflexion strength	Sitting, hip and ankle at 90°. Dorsiflex both feet to end range of motion and hold.	Hold feet in dorsiflexion for 3 × 10 seconds	Increase repetitions up to maximum of 10
Ankle inversion strength	Sitting, hip and ankle at 90°. Invert foot against resistive exercise band anchored by chair leg	3 × 10 repetitions for each foot	Increase resistance strength of resistive exercise band
Ankle eversion strength	Sitting, hip and ankle at 90°. Evert foot against resistive exercise band anchored by chair leg	3 × 10 repetitions for each foot	Increase resistance strength of resistive exercise band
Ankle plantarflexion strength	From standing, rise up on to toes of both feet and back down	3 × 10 repetitions	Increase repetitions up to maximum of 50.
Gastrocnemius stretch	Standing stretch leaning against wall. Stretch leg is extended with knee locked. Support leg forward with knee bent	Hold stretch for 3 × 20 seconds on each leg	Increase forward lean to increase stretch as required
Toe plantarflexion strength	Heel on plate of Archxerciser™. Toes over spring loaded toebar. Retract bar	3 × 10 repetitions for each foot	Increase distance bar is retracted
Toe plantarflexion strength	Pick up 25 mm stones and place in box.	Pick up 2 × 20 stones for each foot.	None
Adductor hallucis stretch	Elastic band around both hallux. Move feet apart	3 × 20 seconds	None

All exercises to be performed 3 times a week for 6 months from baseline to follow up assessment.

(iv) *Falls prevention education*: the booklet "Don't fall for it. Falls can be prevented!" subsidised by the Commonwealth Department of Health and Ageing will be provided. This booklet provides a general overview of risk factors for falls and outlines strategies to prevent falls, including regular eye checks, medication assessment, household hazard assessment and general exercise guidelines.

### Baseline assessments

Demographic data will be collected at baseline and will include age, gender, height, weight, country of birth, education, source of income, falls history, eye problems, health conditions, medications, walking aids, use of community services, foot problems, foot posture and use of podiatry services. The *Active Australia *survey questionnaire will be used to obtain a baseline measurement of the physical activity levels of the participants [42].

### Primary outcome measures

All participants will be followed for 12 months following baseline assessment to record the number of falls. Falls, defined as "an unexpected event in which the participant comes to rest on the ground, floor, or lower level" [43] will be monitored using monthly mail-out calendars. When a fall occurs, specific details about fall injuries will be obtained through structured telephone interviews. If falls calendars are not returned at the end of each month, research staff will contact the participants by telephone to obtain the missing data. Consistent with the recommendations of the Prevention of Falls Network Europe group, three falls outcomes will be used as the primary outcomes: the number of fallers, the number of multiple fallers and the falls rate. As a safety measure, the time to first fall will be recorded as a secondary measure to reflect adverse events from the intervention [43].

### Secondary outcome measures

**Figure 5:**
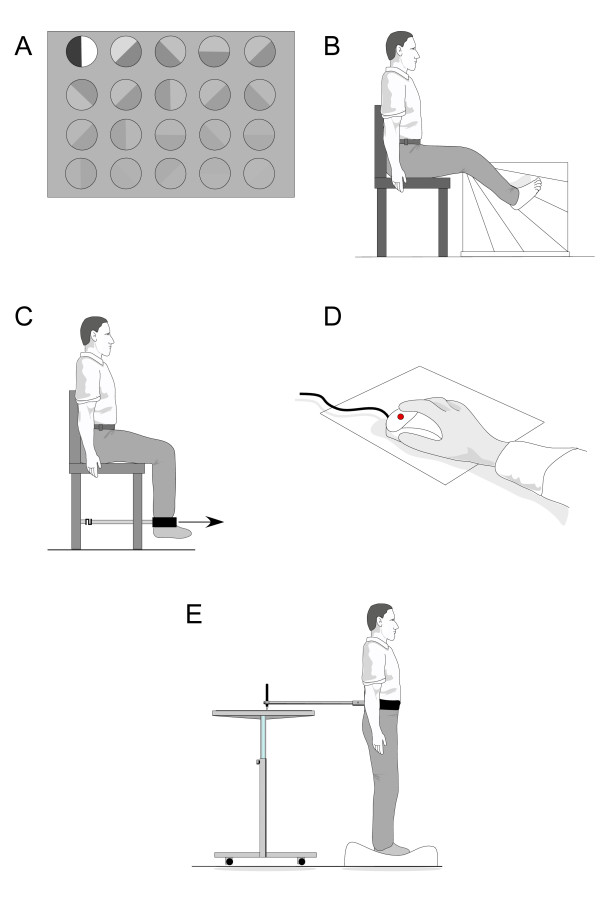
**The short-form Physiological Profile Assessment tests. **A: Visual contrast sensitivity, B: proprioception, C: knee extension strength, D: reaction time, E: postural sway standing on a foam rubber mat.

All participants will be assessed at baseline and at six months by an assessor blinded to group allocation. The secondary outcome measures include:

(i) *Foot pain*: the Manchester Foot Pain and Disability Index (MFPDI) will be used to assess foot pain [39]. The MFPDI consists of 19 statements prefaced by the phrase "Because of pain in my feet", formalised under three constructs: functional limitation (10 items), pain intensity (five items), and personal appearance (two items), with three possible answers: "none of the time" (score = 0), "some days" (score = 1), and "most days/every day" (score = 2). The last two items are concerned with difficulties in performing work or leisure activities, which are omitted if the respondent is of retirement age. The total score (range: 0 to 34) will be used to measure the degree of improvement in foot pain. The MFPDI has been shown to be a suitable tool for assessing foot pain in the older population [44], has high construct validity and has been shown to be sensitive to improvement following a self-management intervention [45].

(ii) *Physiological falls risk*: changes in physiological falls risk will be assessed using the Physiological Profile Assessment (PPA) [5]. Based on the performance of five physiological domains (vision, proprioception, strength, reaction time and balance), the PPA computes a falls risk score (standardised score) for each individual; this measure has a 75% predictive accuracy for falls in older people [46]. The five assessment items (Figure 5) are a test of vision (edge contrast sensitivity using the Melbourne Edge test), peripheral sensation (lower limb proprioception), lower limb strength (knee extension strength), reaction time using a finger press as the response, and body sway (sway when standing on a medium density foam rubber mat). Details regarding each of these tests are provided elsewhere [5]. Several studies have reported significant improvements in PPA scores following exercise interventions [47,48].

(iii) *Fear of falling*: Fear of falling will be measured using the 7-item short Falls Efficacy Scale-International (FES-I) [49]. The FES-I uses a Likert scale to score the participant's level of concern regarding the possibility of falling when performing certain activities of daily living (e.g. taking a bath/shower or climbing up or down stairs). There are four responses which are: "not at all concerned" (score = 1), "somewhat concerned" (score = 2), "fairly concerned" (score = 3) and "very concerned" (score = 4). The total score ranges from 7 (no concern about falling) to 28 (severe concern about falling). The FES-I has been shown to have excellent validity [50] and reliability in the older people (Cronbach's alpha 0.92, ICC = 0.83) [49].

(iv) *Generic health related quality of life*: Mental and physical health status will be measured with the Mental (MCS-12) and Physical (PCS-12) Component Summary scores of the short form Health Survey (SF-12). The SF-12 was developed and validated as a shorter alternative to the SF-36 [51]. To calculate PCS-12 and MCS-12 scores, SF-12 items are scored and normalized via a standardised algorithm. PCS-12 and MCS-12 scores range from 0 to 100, with higher scores indicating better functioning. The MCS-12 and PCS-12 were designed to have a mean score of 50 and a standard deviation of 10 in a representative sample of the US population [52]. Test-retest correlations of 0.89 and 0.76 were observed for the 12-item PCS-12 and the MCS-12, respectively [51].

**Figure 6:**
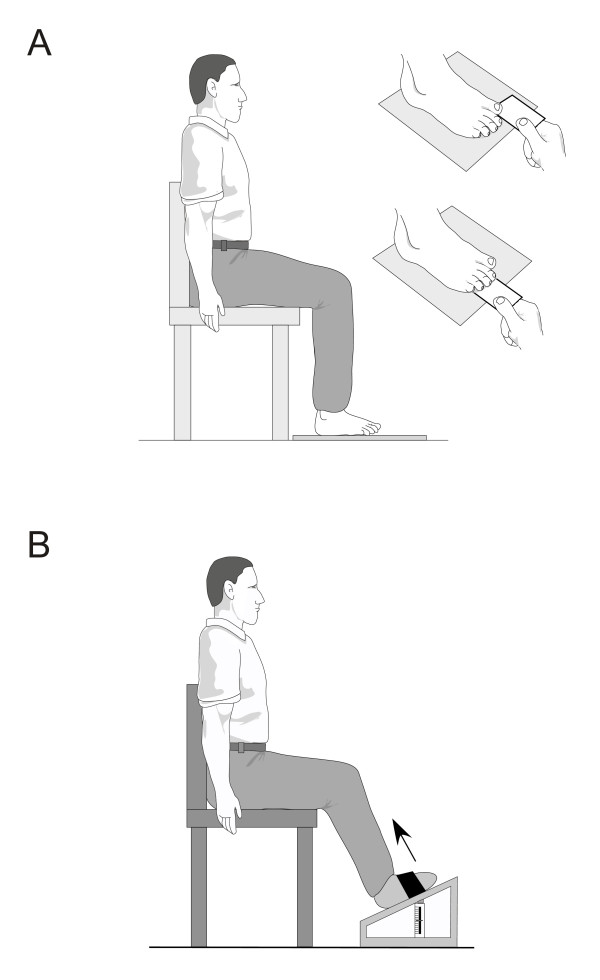
Muscle strength assessment of A: toe plantarflexors, and B: ankle dorsiflexors.

**Figure 7:**
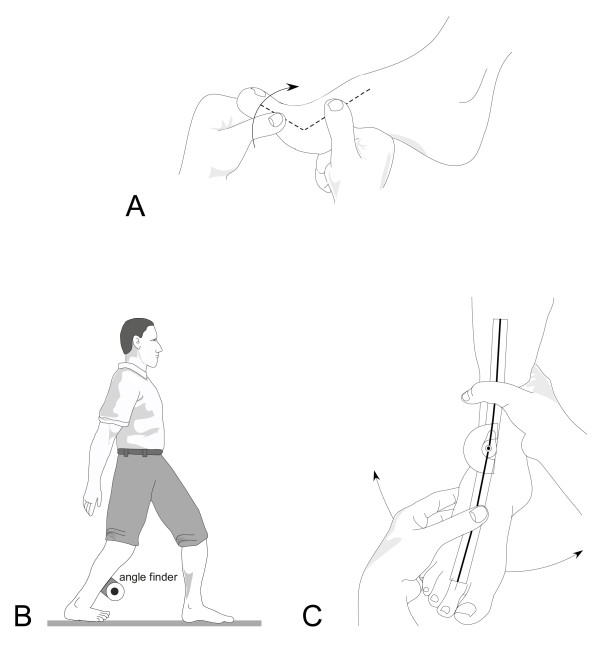
**Assessment of joint range of motion in the foot. **A: first metatarsophalangeal joint, B: ankle joint dorsiflexion, C: ankle joint complex inversion/eversion.

**Figure 8:**
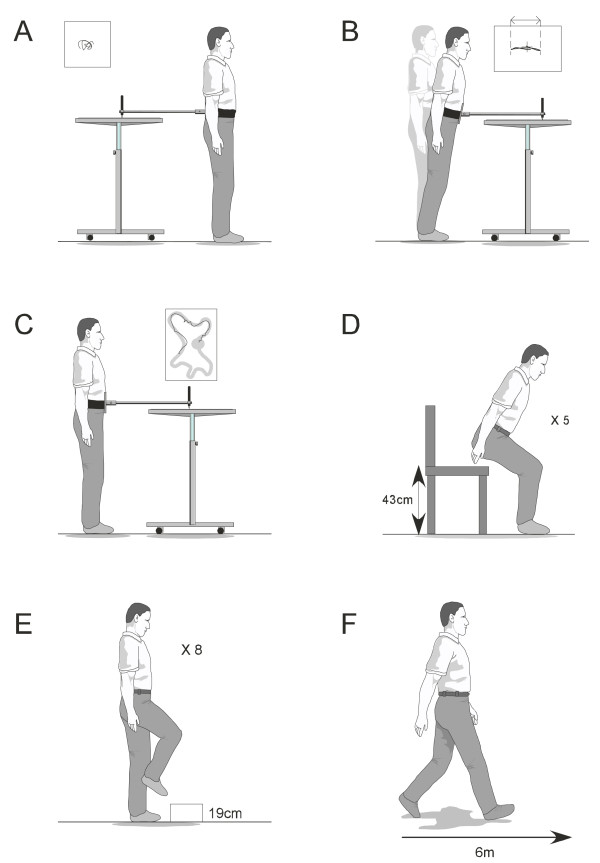
**Balance and functional tests.** A = postural sway, B = maximal balance range, C = coordinated stability, D = sit to stand, E = alternate step test, F = walking speed.

(v) *Foot and ankle strength*: Maximal isometric muscle strength of foot and ankle muscles (ankle dorsiflexion, plantarflexion, inversion and eversion, hallux plantarflexion and lesser toe plantarflexion) will be assessed as the average of three trials using a hand held dynamometer (Citec, CIT Technics, Haren, The Netherlands). Using hand-held dynamometry for testing foot and ankle strength has been shown to have good reliability (ICC 0.88 to 0.95) [53]. Plantarflexion strength of the toes will also be tested using the paper grip test, where the participant is seated with their knee and ankle at 90°, and instructed to use their toe muscles to push down on a 1 mm piece of card (e.g. a business card) while the examiner stabilises their ankle and attempts to slide the card away from the toes (Figure 6A). An inability to hold the card on any one of three trials is recorded as a fail. Using receiver operating characteristics (ROC) curves, this test has been shown to have a positive predictive value of detecting weakness of 95% for the hallux and 90% for the lesser toes [54]. Maximal isometric muscle strength for ankle dorsiflexion will also be measured as the maximum of three trials with the participant sitting in a high chair with their foot secured to a footplate attached to a spring gauge (Figure 6B). This test has been shown to have good reliability (ICC 0.88) [5]. Testing will be conducted on the self-reported dominant side identified by the response to the question "Which foot would you use to kick a ball with?".

(vi) *Foot and ankle range of motion*: First metatarsophalangeal joint dorsiflexion range of motion will be measured using a goniometer as the maximum angle at which the hallux cannot be passively moved into further extension in a non-weightbearing position (Figure 7A). The reliability of this test (ICC 0.95) has been reported previously [55]. Ankle dorsiflexion flexibility will be recorded using a modified version of the lunge test which has been shown to have high reliability (ICC = 0.88) [56]. Participants will be asked to take a comfortable step forward and squat as low as possible, keeping their trunk upright, without lifting the heel of their backfoot from the ground. The degree of motion will be recorded using a digital inclinometer placed on the mid-point of the anterior tibial border (Figure 7B). The test will be conducted with the knee extended and then with the knee flexed [57,58]. To assess ankle inversion and eversion, participants will be seated with the lower leg unsupported. Landmarks will be made on the participants at the midpoint between the malleoli on the anterior aspect of the ankle, the midline on the anterior aspect of the lower leg using the crest of the tibia as a reference point, and the longitudinal midline on the anterior surface of the second metatarsal. A flexible universal goniometer will be aligned along the landmarks and the participants will move their ankle from a self-selected neutral position actively to the end of range of inversion and eversion (Figure 7C). High intra-observer reliability has been reported for these tests (ICC 0.82 to 0.96) [59]. Testing will be conducted on the self-reported dominant side.

(vii) *Balance and functional ability*: Balance will be assessed through four tests. Postural sway will be measured as previously described in the section regarding the PPA (Figure 8A). The reliability (ICC 0.57 to 0.68) of this test has been reported previously [60]. Lateral stability will be measured using a sway meter that measures displacement of the body at the waist with testing performed with the participant standing with one foot in front of the other. Participants will choose which foot to place in the forward position and will be instructed to stand as still as possible for 30 seconds. Lateral displacement of the body will then be recorded [61]. Leaning balance will be measured using the maximum balance range test and coordinated stability tests [62]. For the maximum balance test, (Figure 8B) the sway meter is attached around the waist with the rod extending anteriorly. Participants are asked to lean forward then backwards from the ankles without moving their feet, as far as possible, i.e. to the point where they can just retain their balance. Maximal anterior-posterior displacement is measured over three trials. Using similar apparatus, the coordinated stability test (Figure 8C) requires the participant to bend and rotate at the hips without moving their feet to move the sway meter pen around a convoluted track marked on a piece of paper attached to the top of an adjustable height table. A total error score is calculated by summing the number of occasions the pen strays outside the track [62]. Good reliability has been reported for both the maximum balance test (ICC = 0.74) and the coordinated stability test (ICC = 0.83) [62] and both tests are predictive of falls[63]. These tests will be performed twice, once with the participant barefoot and again with their preferred outdoor footwear. Lateral stability, maximum balance range, coordinated stability and walking speed will be corrected for height prior to analysis [11].

Functional ability will be evaluated using the sit to stand test (time taken to rise from a 43 cm high chair five times without using the arms) (Figure 8D), alternate stepping test (the time taken to alternately place each foot on a 19 cm high step eight times) (Figure 8E), and walking speed over 6 metres (Figure 8F). These tests have been shown to be reliable in previous studies of older people (sit to stand (ICC = 0.89) [64], alternate stepping test (reliability coefficient of 0.98) [65] and 6 m walk (reliability coefficient of 0.93) [66] and have demonstrated good sensitivity and specificity in identifying multiple fallers [38].

### Sample size

The sample size for the study has been determined *a priori *using an appropriate sample size formula [67]. A sample size of 143 participants in each group provides 80% power to detect a 30% reduction in the intervention group in the primary outcome of the percentage of fallers. This reduction in falls was based on the following: (i) a falling rate of 60% in the control group (as has been observed in older people with foot pain in a previous risk factor study by Menz et al [12]), and (ii) a falling rate of 42% in the intervention group (which equates to a 30% reduction in the number of fallers, recently demonstrated in a similar multifaceted study by Clemson et al [68]). We chose an alpha level of 0.05 and allowed for a drop-out rate of 15%. To allow for unforeseen circumstances we will aim to recruit 300 participants (i.e. 150 per group).

### Statistical analysis

Statistical analysis will be undertaken using SPSS version 14.0 (SPSS Corp, Chicago, Ill, USA) and STATA 8 (Stata Corp, College Station, Tex., USA) statistical software. All analyses will be conducted on an intention-to-treat principle using all randomised participants [69]. Missing data will be replaced with the last score carried forward. Demographic characteristics and baseline data will be summarised by descriptive statistics. Consistent with the recommendations of the Prevention of Falls Network Europe group [43], three falls outcomes will be used as the primary outcomes: the number of fallers, the number of multiple fallers and the falls rate. The number of fallers and multiple fallers (two or more falls) will be compared by calculating relative risks. The number of falls and falls rate per person per year in the two groups will then be compared using negative binomial regression models. This approach takes into account all falls and adjusts for varying duration of follow-up [70,71]. The continuously-scored secondary outcome measures at baseline and the six month follow-up appointments will be compared using analysis of covariance with baseline scores and intervention group entered as independent variables [72,73].

## Discussion

This study is a randomised trial designed to investigate whether a multifaceted podiatry intervention can prevent falls, enhance balance and reduce disabling foot pain in older people. It will report on all the outcome measures recommended by Prevention of Falls Network Europe, a collaborative project to promote best practice in research in falls in older people. These outcome measures are falls, fall injury, physical activity, psychological consequences, and generic health related quality of life [43].

While current guidelines [20,32] generally recommend multifactorial interventions, a recent meta-analysis has highlighted that a single targeted intervention can be as effective as multifactorial fall prevention programs [74]. The current study targets podiatry interventions using a multifaceted approach. The trial interventions are all management strategies commonly used by podiatrists for treatment of foot-related problems in the general population. While there is some evidence that these interventions can improve balance, this is the first trial to combine specific foot and shoe-related interventions in a falls prevention trial.

A key factor in determining the efficacy of a falls prevention intervention is the degree of adherence to the program. While we do not expect any difficulties to arise in relation to the "usual care" component of the study, adherence may be more difficult to achieve in relation to the provision of new footwear and the home-based exercise program. Indeed, previous studies have indicated that compliance with use of therapeutic footwear has been shown to be low in people with diabetes [75] and rheumatoid arthritis [76] and a recent survey of emergency department physicians indicated that compliance with footwear recommendations to prevent falls was poor, due to "stubbornness and vanity" [77]. However, a recent evaluation of people attending falls clinics reported that 85% had partly or fully complied with footwear advice, suggesting that older people who are aware of their risk of future falls may be more likely to adhere to footwear recommendations [78].

In a review of 21 randomised trials of exercise including middle and older aged adults, the average rate of adherence to exercise training (typically measured by frequency of exercise) was reported to be 78%, reducing to 63% when data from participants who dropped out of the exercise intervention was included [79]. However, there is evidence that strength training can be successful when undertaken at home alone [80] and that many older people prefer to exercise at home [81]. The use of exercise sheets with written descriptions and diagrams [82], training diaries [83] and continued support through either mail or telephone contact from the exercise instructor [84] have all been shown to increase adherence. To optimise adherence in our trial, we intend to utilise each of these strategies.

In summary, this project is the first randomised trial to be conducted to evaluate the efficacy of podiatry in improving balance and preventing falls in older people. The intervention has been pragmatically designed to ensure that the study findings can be implemented into clinical practice if found to be an effective falls prevention strategy. Recruitment for the study will commence in July 2008, and we expect final results to be available in mid 2011.

## Competing interests

The authors declare that they have no competing interests.

## Authors' contributions

HBM and SRL conceived the idea and obtained funding for the study. MJS, HBM and SRL designed the trial protocol. MJS and HBM drafted the manuscript. All authors have read and approved the final manuscript.

## Pre-publication history

The pre-publication history for this paper can be accessed here:


